# Supplementary material for: Unraveling the energy storage mechanism in graphene-based nonaqueous electrochemical capacitors by gap-enhanced Raman spectroscopy
Source: Nat Commun. 2024 Jul 4;15:5624. doi: 10.1038/s41467-024-49973-9 (PMC11224393; doi:10.1038/s41467-024-49973-9)
Supplement: Supplementary file 1 — Supplementary Information [file 41467_2024_49973_MOESM1_ESM.pdf]

Supplementary information

Unraveling the energy storage mechanism in graphene-based nonaqueous electrochemical capacitors by gap-enhanced Raman spectroscopy

Xiao-Ting Yin[1], En-Ming You[2], Ru-Yu Zhou[1], Li-Hong Zhu[3], Wei-Wei Wang[1], Kai-Xuan Li[1], De-Yin Wu[1], Yu Gu[1*], Jian-Feng Li[1*], Bing-Wei Mao[1], Jia-Wei Yan[1*]

[1]State Key Laboratory of Physical Chemistry of Solid Surfaces, College of Chemistry and Chemical Engineering, Xiamen University, Xiamen 361005, China

[2]School of Ocean Information Engineering, Fujian Provincial Key Laboratory of Oceanic Information Perception and Intelligent Processing, Jimei University, Xiamen 361021, China

[3]Department of Electronic Science, Xiamen University, Xiamen 361005, China

*e-mail: jwyan@xmu.edu.cn; li@xmu.edu.cn; ygu@xmu.edu.cm

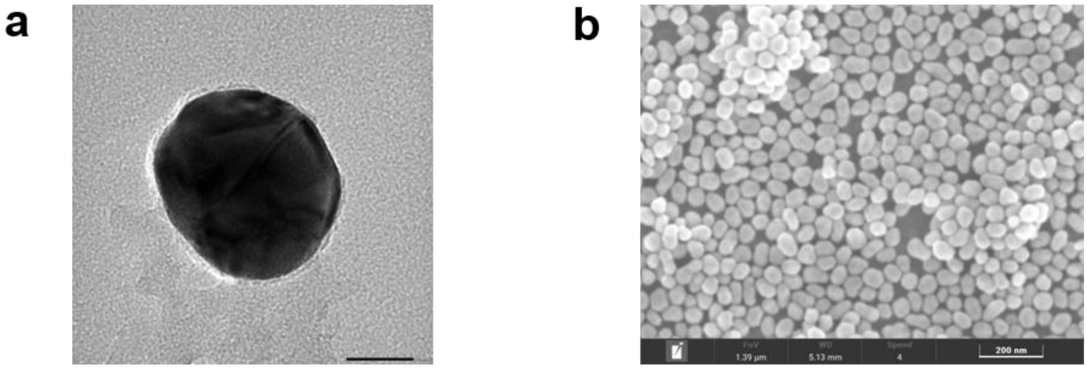

Supplementary Figure 1. SHINs images (a) TEM image of an $Au@SiO_2$ nanoparticle. Scale bar: 20 nm. **(b)** SEM image of SHINs distribution on graphene. Scale bar: 200 nm.

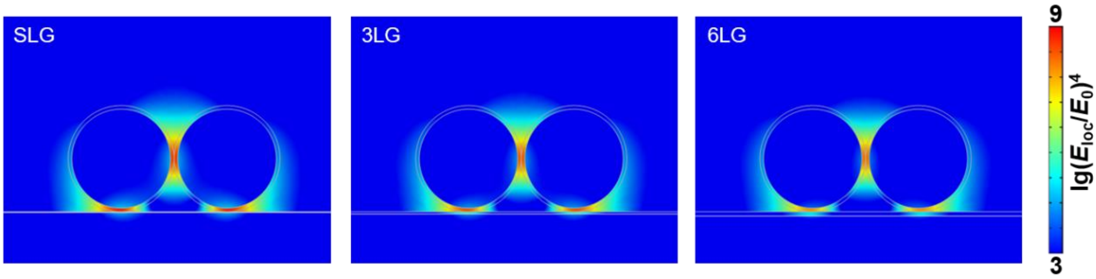

Supplementary Figure 2. Electromagnetic field distribution around $Au@SiO_2$/graphene/Cu simulated by COMSOL finite element method.

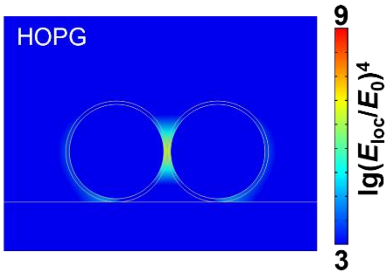

Supplementary Figure 3. Electromagnetic field distribution around $Au@SiO_2$/HOPG simulated by COMSOL finite element method.

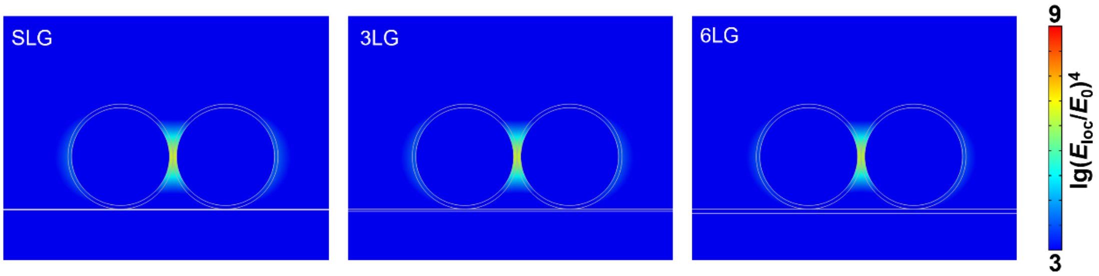

Supplementary Figure 4. Electromagnetic field distribution around Au@SiO_2/graphene/SiO_2 simulated by COMSOL finite element method.

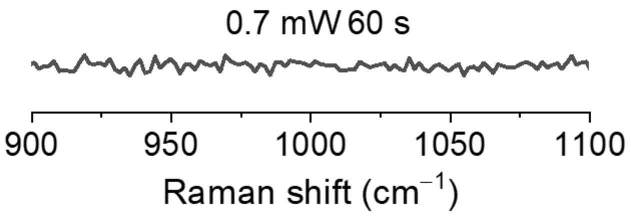

Supplementary Figure 5. Raman spectra of Au film in 0.01 M pyridine (Py). Laser power was controlled at about 0.7 mW and accumulation time was set to 60 s**.**

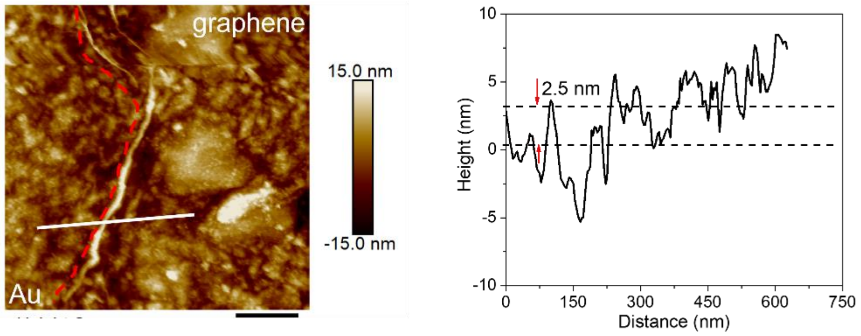

Supplementary Figure 6. AFM image of six-layer graphene on a Au substrate (with the red dashed line indicating the boundary) and sectional analysis of the white line in the image for measuring the height of the transferred graphene. Scale bar: 300 nm.

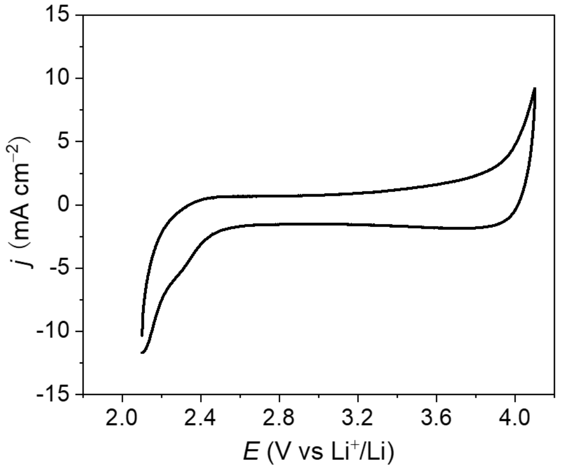

Supplementary Figure 7. Cyclic voltammogram of HOPG. Scan rate: 10 mV s^{-1}.

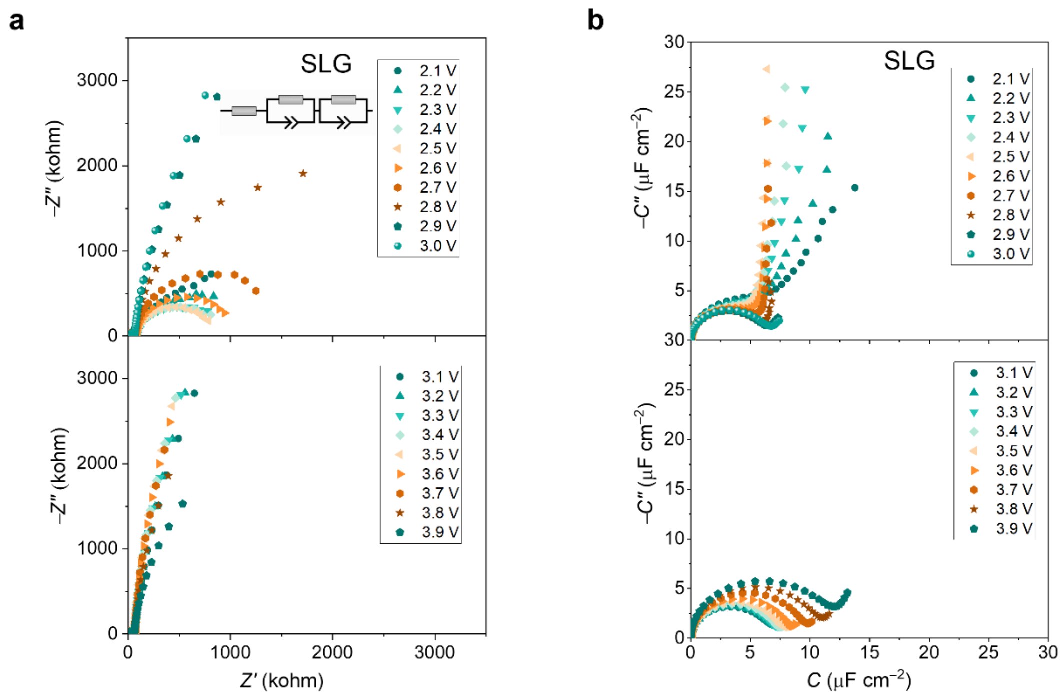

Supplementary Figure 8. Nyquist plots **(a)** and complex capacitance plots **(b)** of SLG at different potentials.

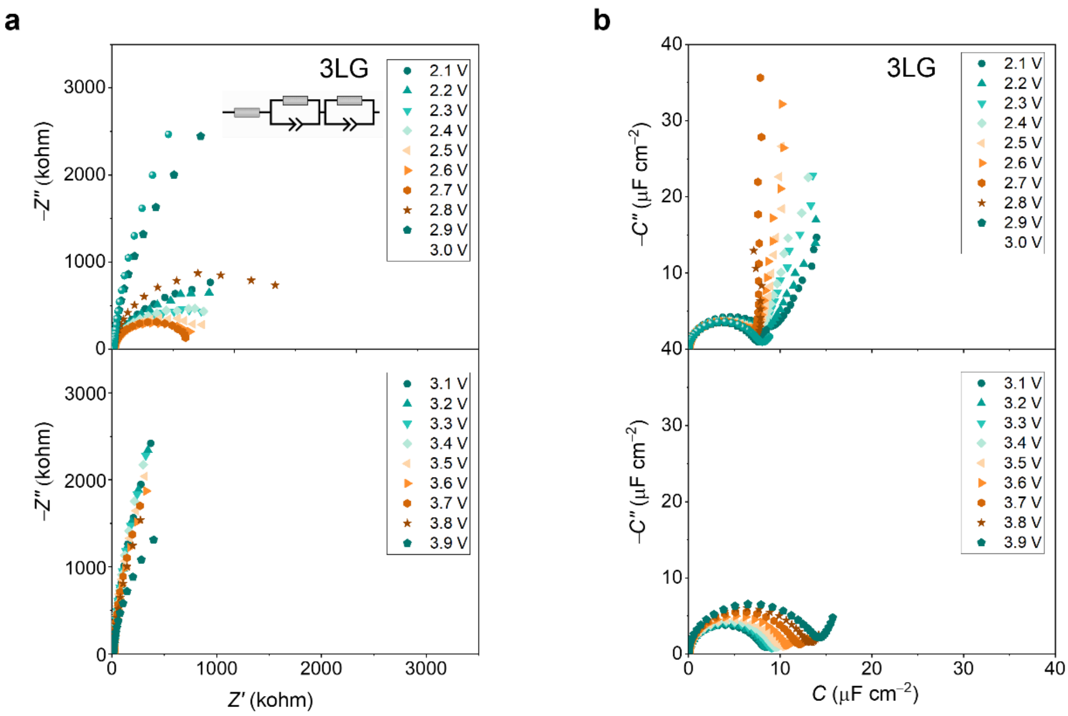

Supplementary Figure 9. Nyquist plots **(a)** and complex capacitance plots **(b)** of 3LG at different potentials.

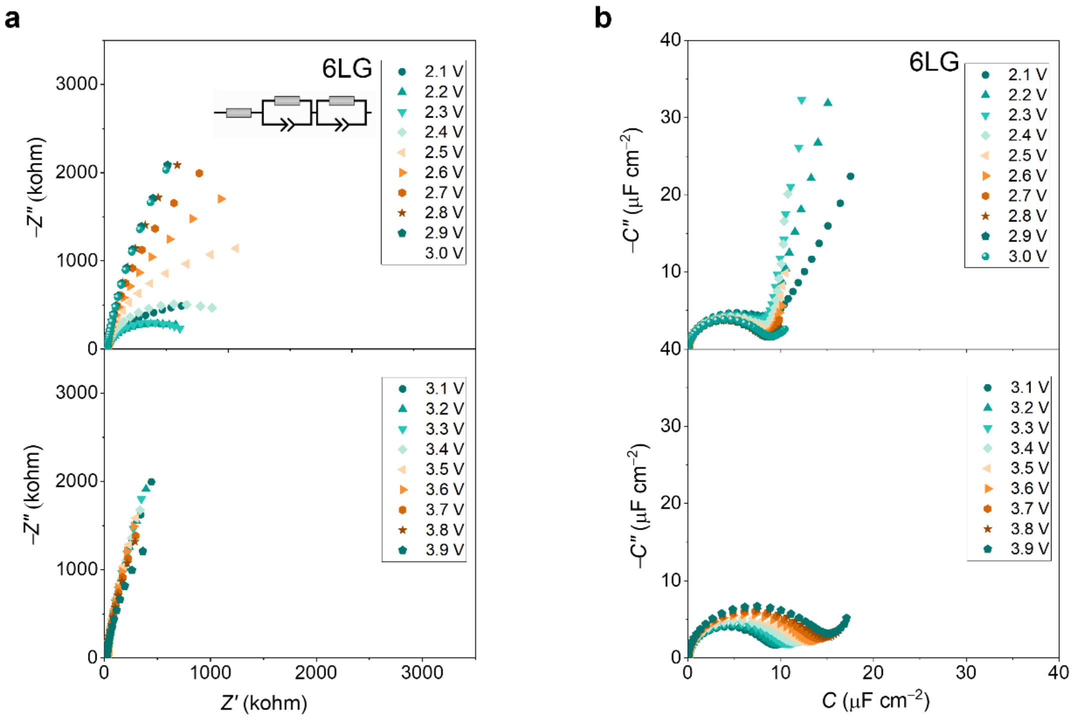

Supplementary Figure 10. Nyquist plots **(a)** and complex capacitance plots **(b)** of 6LG at different potentials.

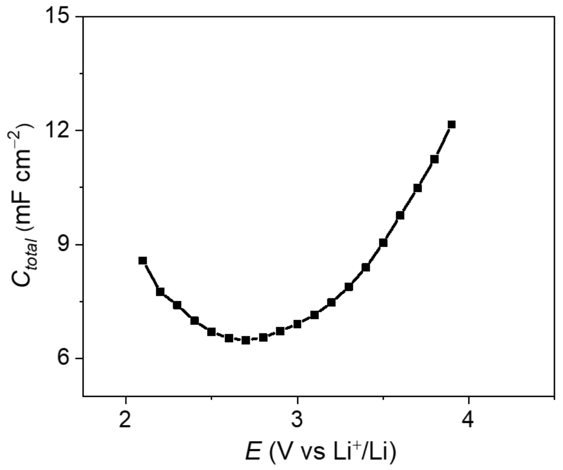

Supplementary Figure 11. Differential capacitance curve of HOPG.

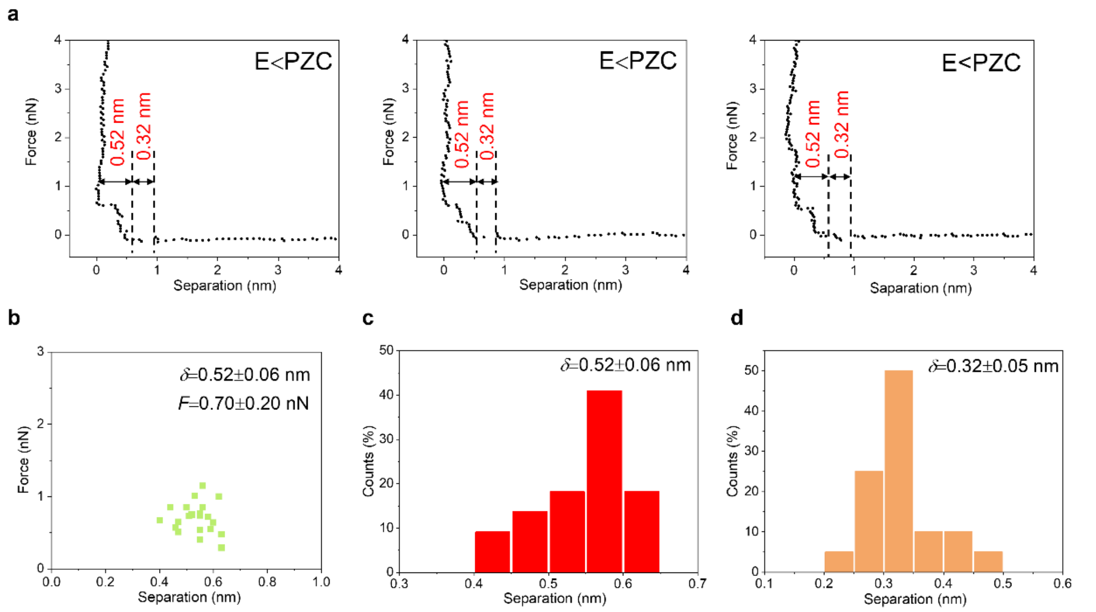

Supplementary Figure 12. (a) Three AFM force curves when E<PZC. **(b)** The distribution of the first layer thickness and force value when E<PZC. **(c)** and **(d)** Two-dimensional histograms of the first layer thickness and the second layer thickness when E<PZC.

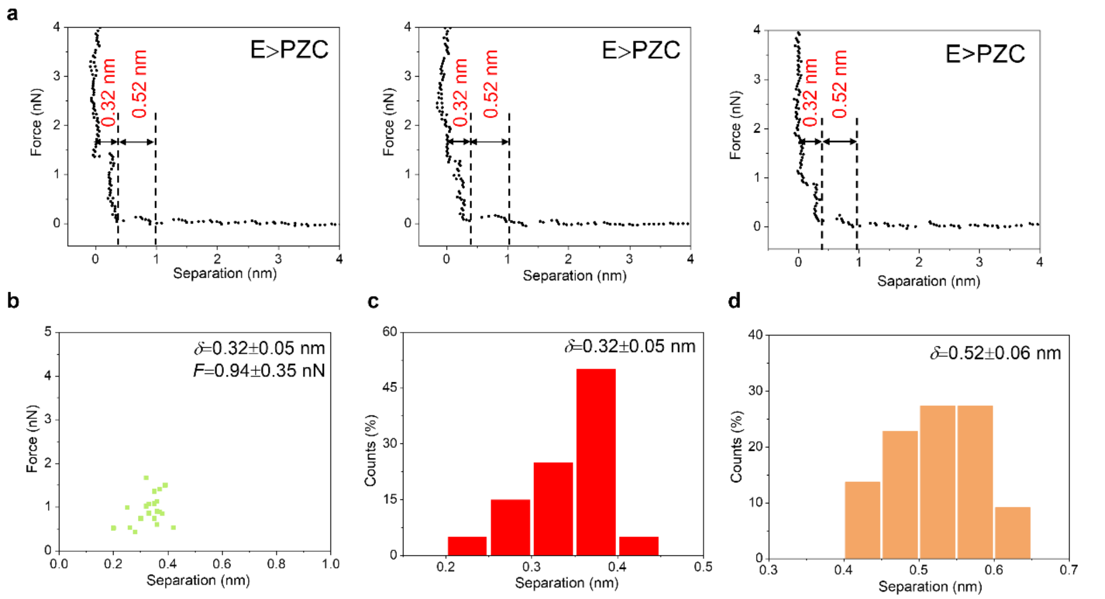

Supplementary Figure 13. (a) Three AFM force curves when E>PZC. **(b)** The distribution of the first layer thickness and force value when E>PZC. **(c)** and **(d)** Two-dimensional histograms of the first layer thickness and the second layer thickness when E>PZC.

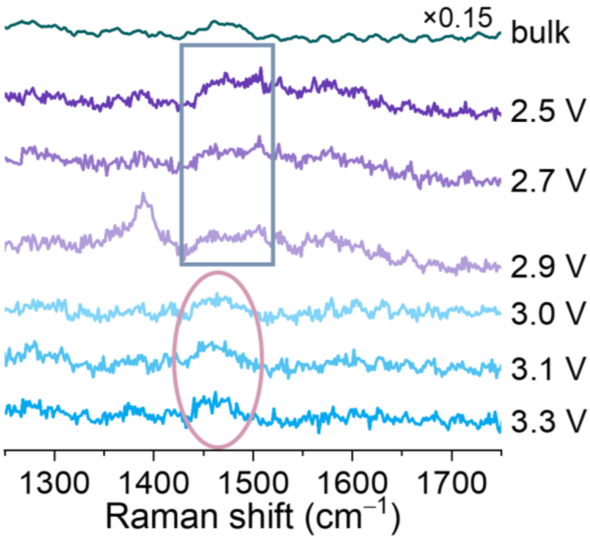

Supplementary Figure 14. CH_2 bending/scissoring mode.

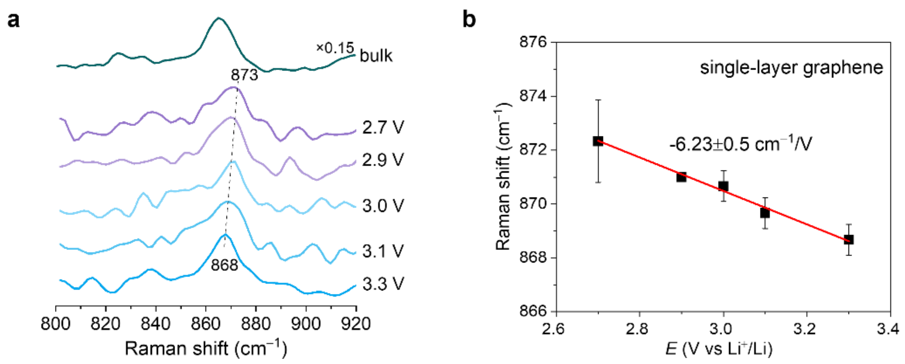

Supplementary Figure 15. (a) The spectra of the crown ether-like breathing mode of $Li(G4)^+$, which were smoothed using second-order processing with LabSpec software. **(b)** The Raman shifts of crown ether-like breathing mode of $Li(G4)^+$ on single-layer graphene as a function of electrode potential. Error bars represent s.d. for each data point (n = 3 independent experiments), and points are average values.

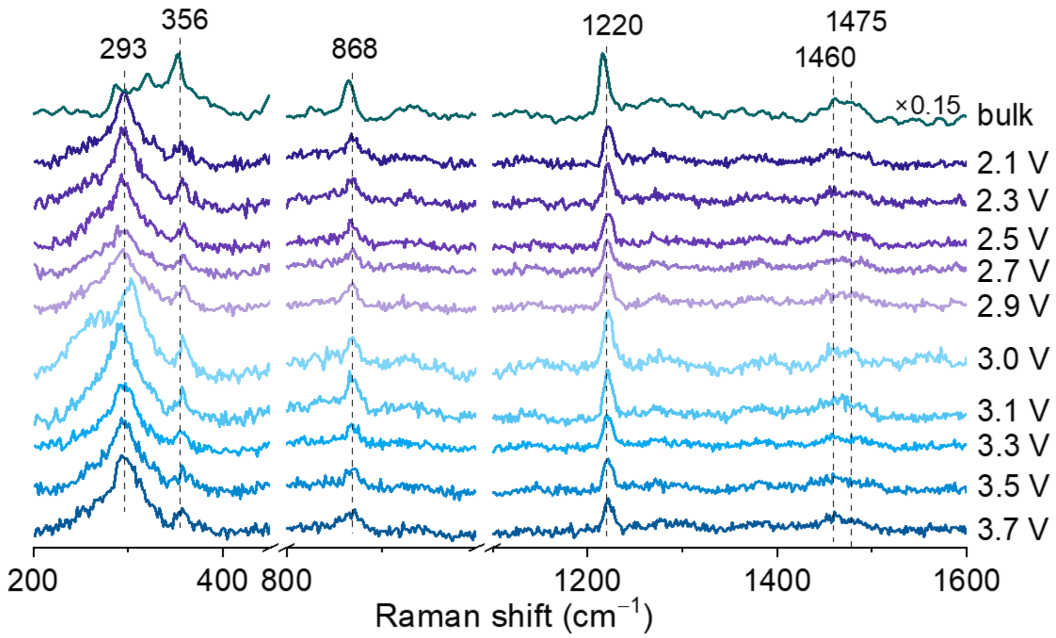

Supplementary Figure 16. In-situ SHINERS spectra of [Li(G4)][[FSI] on $Au@SiO_2$/graphite

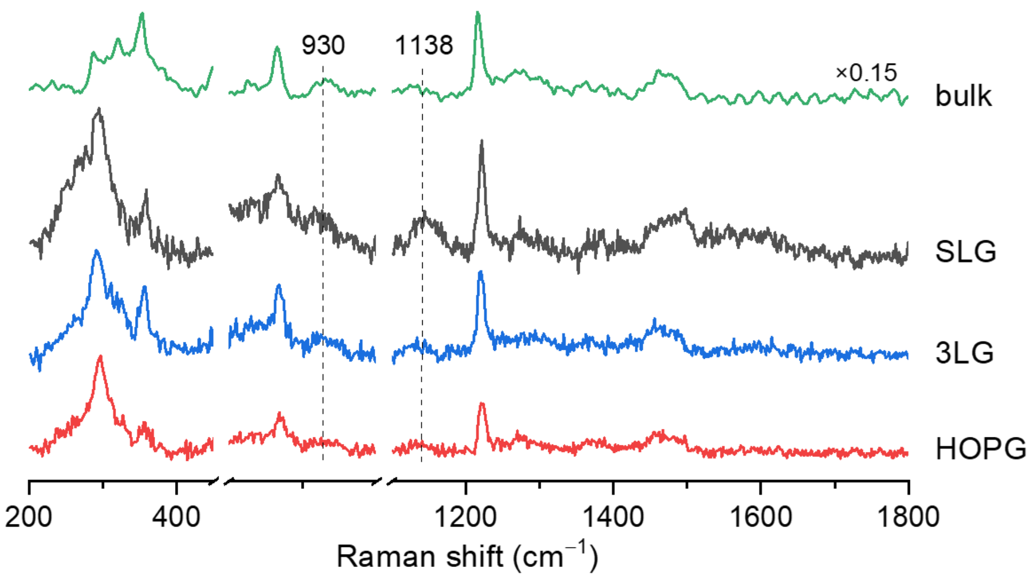

Supplementary Figure 17. Raman spectra of the electrolyte adsorbates at the interface for single-layer graphene, three-layer graphene, and graphite configurations at 2.1 V (vs. Li^+/Li).

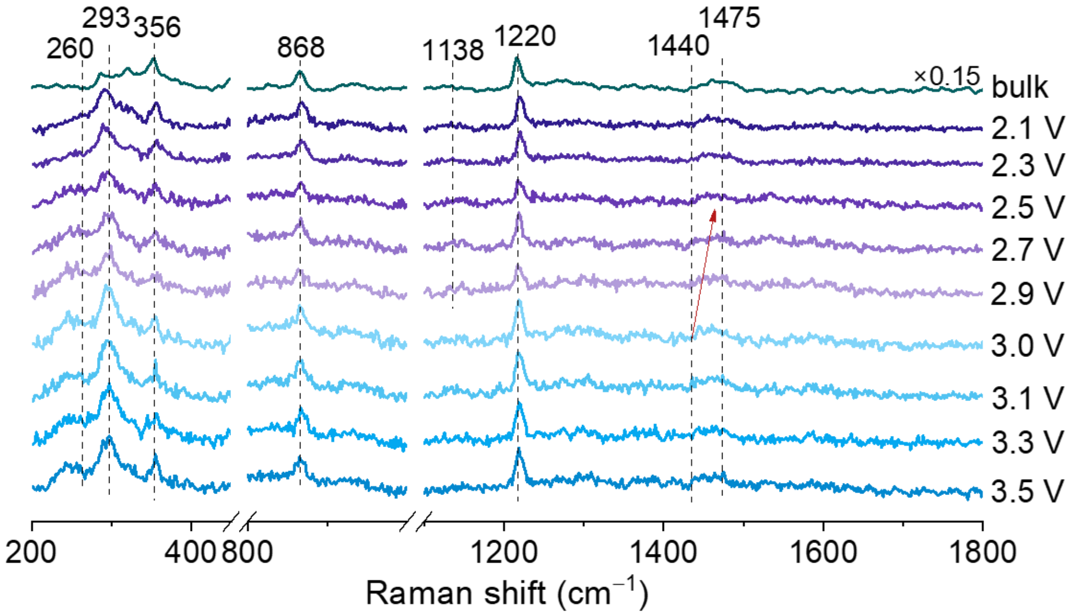

Supplementary Figure 18. In-situ SHINERS spectra of [Li(G4)][[FSI] on Au@SiO_2/three-layer graphene/Au.

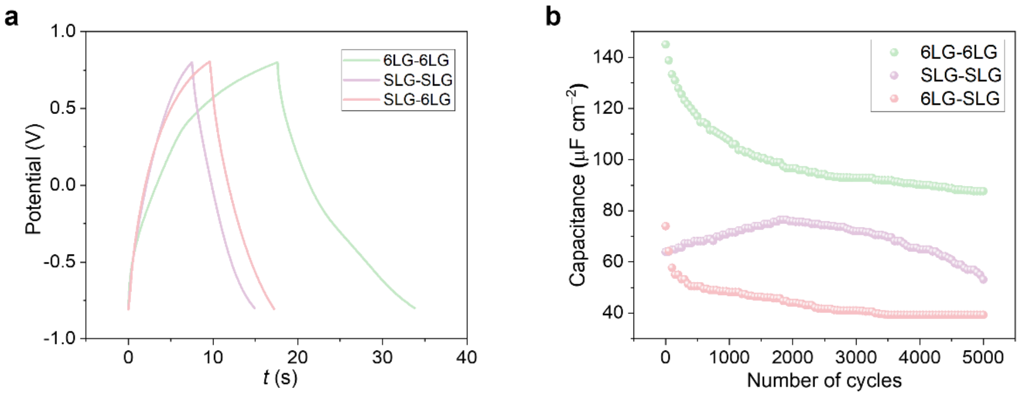

Supplementary Figure 19. Measurements of the symmetric supercapacitor in [Li(G4)][[FSI] up to 5000 cycles.

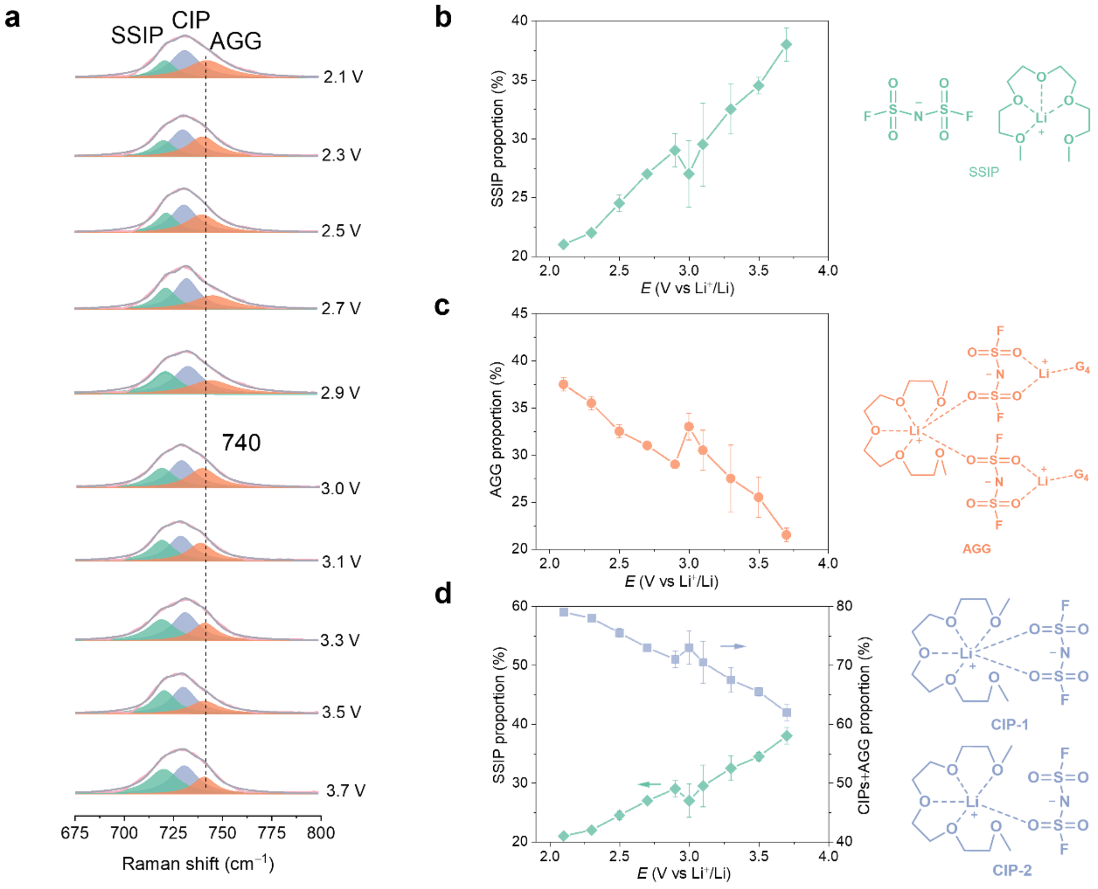

Supplementary Figure 20. (a) Potential-dependent Raman spectra of coordination structures at six-layer graphene interface. **(b-d)** Potential-dependent contents of SSIP, CIPs and AGG at six-layer graphene interface.

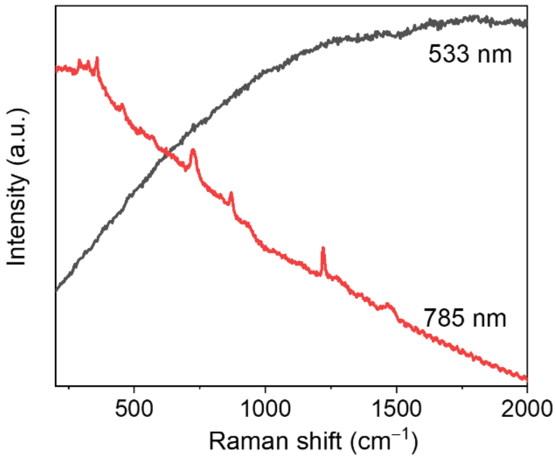

Supplementary Figure 21. Raman spectra of [Li(G4)][[FSI] under different excitation laser.

Supplementary Table 1. Comparison of the performance of electrochemical capacitor based on graphene electrodes.

Electrode (graphene)	C_m (F g^{-1})	E (Wh kg^{-1})
single-layer/single-layer	97	34
six-layer/six-layer	220	78
